# 121 An Examination of Function at Follow-Up in Historically Marginalized Persons with Burn Injury

**DOI:** 10.1093/jbcr/irad045.094

**Published:** 2023-05-15

**Authors:** Betsey Ferreira, Lynne Benavides, Miranda Yelvington, Kathryn Lundquist, Lauren J Shepler, Jeffrey Schneider, Kyra Jeanine Solis, Samuel Mandell, Nicole Gibran, Kara McMullen

**Affiliations:** Rhode Island Hospital, Cumberland, Rhode Island; Rhode Island Burn Center, Hope Valley, Rhode Island; Arkansas Children's Hospital, Little Rock, Arkansas; University of Iowa Hospitals & Clinics, North Liberty, Iowa; Spaulding Rehabilitation Hospital, Harvard Medical School, Boston, Massachusetts; Spaulding Rehabilitation Hospital/Harvard Medical School/Rehabilitation Outcomes Center at Spaulding, Boston, Massachusetts; UT Southwestern Medical Center, Dallas, Texas; Parkland Burn Center / UT Southwestern, Dallas, Texas; University of Washington, Seattle, Washington; University of Washington, Seattle, Washington; Rhode Island Hospital, Cumberland, Rhode Island; Rhode Island Burn Center, Hope Valley, Rhode Island; Arkansas Children's Hospital, Little Rock, Arkansas; University of Iowa Hospitals & Clinics, North Liberty, Iowa; Spaulding Rehabilitation Hospital, Harvard Medical School, Boston, Massachusetts; Spaulding Rehabilitation Hospital/Harvard Medical School/Rehabilitation Outcomes Center at Spaulding, Boston, Massachusetts; UT Southwestern Medical Center, Dallas, Texas; Parkland Burn Center / UT Southwestern, Dallas, Texas; University of Washington, Seattle, Washington; University of Washington, Seattle, Washington; Rhode Island Hospital, Cumberland, Rhode Island; Rhode Island Burn Center, Hope Valley, Rhode Island; Arkansas Children's Hospital, Little Rock, Arkansas; University of Iowa Hospitals & Clinics, North Liberty, Iowa; Spaulding Rehabilitation Hospital, Harvard Medical School, Boston, Massachusetts; Spaulding Rehabilitation Hospital/Harvard Medical School/Rehabilitation Outcomes Center at Spaulding, Boston, Massachusetts; UT Southwestern Medical Center, Dallas, Texas; Parkland Burn Center / UT Southwestern, Dallas, Texas; University of Washington, Seattle, Washington; University of Washington, Seattle, Washington; Rhode Island Hospital, Cumberland, Rhode Island; Rhode Island Burn Center, Hope Valley, Rhode Island; Arkansas Children's Hospital, Little Rock, Arkansas; University of Iowa Hospitals & Clinics, North Liberty, Iowa; Spaulding Rehabilitation Hospital, Harvard Medical School, Boston, Massachusetts; Spaulding Rehabilitation Hospital/Harvard Medical School/Rehabilitation Outcomes Center at Spaulding, Boston, Massachusetts; UT Southwestern Medical Center, Dallas, Texas; Parkland Burn Center / UT Southwestern, Dallas, Texas; University of Washington, Seattle, Washington; University of Washington, Seattle, Washington; Rhode Island Hospital, Cumberland, Rhode Island; Rhode Island Burn Center, Hope Valley, Rhode Island; Arkansas Children's Hospital, Little Rock, Arkansas; University of Iowa Hospitals & Clinics, North Liberty, Iowa; Spaulding Rehabilitation Hospital, Harvard Medical School, Boston, Massachusetts; Spaulding Rehabilitation Hospital/Harvard Medical School/Rehabilitation Outcomes Center at Spaulding, Boston, Massachusetts; UT Southwestern Medical Center, Dallas, Texas; Parkland Burn Center / UT Southwestern, Dallas, Texas; University of Washington, Seattle, Washington; University of Washington, Seattle, Washington; Rhode Island Hospital, Cumberland, Rhode Island; Rhode Island Burn Center, Hope Valley, Rhode Island; Arkansas Children's Hospital, Little Rock, Arkansas; University of Iowa Hospitals & Clinics, North Liberty, Iowa; Spaulding Rehabilitation Hospital, Harvard Medical School, Boston, Massachusetts; Spaulding Rehabilitation Hospital/Harvard Medical School/Rehabilitation Outcomes Center at Spaulding, Boston, Massachusetts; UT Southwestern Medical Center, Dallas, Texas; Parkland Burn Center / UT Southwestern, Dallas, Texas; University of Washington, Seattle, Washington; University of Washington, Seattle, Washington; Rhode Island Hospital, Cumberland, Rhode Island; Rhode Island Burn Center, Hope Valley, Rhode Island; Arkansas Children's Hospital, Little Rock, Arkansas; University of Iowa Hospitals & Clinics, North Liberty, Iowa; Spaulding Rehabilitation Hospital, Harvard Medical School, Boston, Massachusetts; Spaulding Rehabilitation Hospital/Harvard Medical School/Rehabilitation Outcomes Center at Spaulding, Boston, Massachusetts; UT Southwestern Medical Center, Dallas, Texas; Parkland Burn Center / UT Southwestern, Dallas, Texas; University of Washington, Seattle, Washington; University of Washington, Seattle, Washington; Rhode Island Hospital, Cumberland, Rhode Island; Rhode Island Burn Center, Hope Valley, Rhode Island; Arkansas Children's Hospital, Little Rock, Arkansas; University of Iowa Hospitals & Clinics, North Liberty, Iowa; Spaulding Rehabilitation Hospital, Harvard Medical School, Boston, Massachusetts; Spaulding Rehabilitation Hospital/Harvard Medical School/Rehabilitation Outcomes Center at Spaulding, Boston, Massachusetts; UT Southwestern Medical Center, Dallas, Texas; Parkland Burn Center / UT Southwestern, Dallas, Texas; University of Washington, Seattle, Washington; University of Washington, Seattle, Washington; Rhode Island Hospital, Cumberland, Rhode Island; Rhode Island Burn Center, Hope Valley, Rhode Island; Arkansas Children's Hospital, Little Rock, Arkansas; University of Iowa Hospitals & Clinics, North Liberty, Iowa; Spaulding Rehabilitation Hospital, Harvard Medical School, Boston, Massachusetts; Spaulding Rehabilitation Hospital/Harvard Medical School/Rehabilitation Outcomes Center at Spaulding, Boston, Massachusetts; UT Southwestern Medical Center, Dallas, Texas; Parkland Burn Center / UT Southwestern, Dallas, Texas; University of Washington, Seattle, Washington; University of Washington, Seattle, Washington; Rhode Island Hospital, Cumberland, Rhode Island; Rhode Island Burn Center, Hope Valley, Rhode Island; Arkansas Children's Hospital, Little Rock, Arkansas; University of Iowa Hospitals & Clinics, North Liberty, Iowa; Spaulding Rehabilitation Hospital, Harvard Medical School, Boston, Massachusetts; Spaulding Rehabilitation Hospital/Harvard Medical School/Rehabilitation Outcomes Center at Spaulding, Boston, Massachusetts; UT Southwestern Medical Center, Dallas, Texas; Parkland Burn Center / UT Southwestern, Dallas, Texas; University of Washington, Seattle, Washington; University of Washington, Seattle, Washington

## Abstract

**Introduction:**

Previous studies demonstrate that historically marginalized individuals living with burn injury received comparable physical and occupational therapy services to non-marginalized groups. However, other outcomes are not well studied in these groups. This study aims to examine functional outcomes of historically marginalized burn population.

**Methods:**

Data was obtained from the Burn Model System National Database between 1993 and 2015 for adults alive at discharge. The marginalized group was defined as: age >65; non-White; Hispanic; Medicaid or public support/indigent; homeless; drug or alcohol misuse prior to injury. Demographic and clinical characteristics were examined. Function was compared between groups (marginalized vs. non-marginalized) using the SF-12/VR-12 Physical (PCS) and Mental Component Summary (MCS) Scores at 6 and 12 months after injury.

**Results:**

The sample included 583 participants (69.3%, n=404 marginalized group), was 83.9% white, and mean burn size was 16.7% (SD 17.6) total body surface area. Follow-up status was not significantly different between groups at 12 (p=0.115) and 24 (p=0.250) months. The non-marginalized group had higher PCS and MCS scores at 6 months than the marginalized group (Table). There were no significant differences in outcomes between groups at other time points.

**Conclusions:**

The marginalized group exhibited worse physical and mental function scores than the non-marginalized group. This suggests that despite similar follow-up rates marginalized populations have worse outcomes.

**Applicability of Research to Practice:**

Differences in functional scores between groups suggest that patients from historically marginalized groups may require more resources to achieve similar outcomes to their non-marginalized counterparts. Further research is necessary to delineate the unique needs of historically marginalized groups and design interventions to achieve equitable outcomes.

